# Targeting oxidative stress-NLRP3-apoptotic-steroidogenic axis in doxorubicin-induced testicular toxicity: protective efficacy of ferulic acid niosomes

**DOI:** 10.1186/s40659-026-00707-9

**Published:** 2026-07-27

**Authors:** Mahran Mohamed Abd El-Emam, Aya Elhady Atia, Mona A. Bakry, Rabab A. Husseini, Eman Elrefaei, Noha Osama El-Shaer, Safaa I. Khater, Ola Ali, Ahmed El-Sayed Yousef, Hussein I. Elbelbesy

**Affiliations:** 1 Department of Biochemistry and Molecular Biology, Faculty of Veterinary Medicine, Zagazig University, Zagazig, 44511 Egypt; 2 Department of Physiology, Faculty of Veterinary Medicine, Zagazig University, Zagazig, 44511 Egypt; 3 Department of Pharmaceutics, Faculty of Pharmacy, Zagazig University, Zagazig, 44519 Egypt; 4 Department of Medical Biochemistry, Faculty of Medicine, Tanta University, Tanta, Egypt; 5 Department of Physiology, Faculty of Medicine, Benha University, Banha, 13518 Egypt; 6 Department of Basic Medical Sciences, College of Medicine, Almaarefa University, 13713 Dariyah, Saudi Arabia; 7 Research Center, Deanship of Scientific Research and Postgraduate Studies, Almaarefa University, 13713 Dariyah, Saudi Arabia; 8 Department of Anatomy and Embryology, Faculty of Medicine, Al-Azhar University, Cairo, Egypt

**Keywords:** Doxorubicin, Testicular toxicity, Oxidative stress, NLRP3 inflammasome, Steroidogenesis, Ferulic acid–loaded niosomes

## Abstract

Doxorubicin (DOX) is a commonly prescribed chemotherapeutic agent whose clinical application is limited by its toxicity to rapidly dividing organs, particularly the testes.This study explored the protective potential of ferulic acid-loaded niosomes (FA-NIO) against DOX-induced testicular dysfunction in rats and elucidated the underlying molecular mechanisms. Forty male rats were allocated into four groups: Group 1 was given saline, Group 2 was given DOX, Group 3 was given DOX + free FA, and Group 4 was given DOX + FA-NIO. Serum reproductive hormone levels, testicular oxidative stress indices, and the expression of genes and proteins relevant to NLRP3 inflammasome signaling, apoptosis (Bax and Bcl-2), and steroidogenesis (StAR, CYP11A1, and 3β-HSD) were assessed, along with histopathological changes in the testicular tissue. DOX administration significantly increased MDA (malondialdehyde) while suppressing antioxidant defences, accompanied by hormonal imbalance, severe histopathological damage, upregulation of Bax, NLRP3, and IL-1β, downregulation of Bcl-2, and inhibition of steroidogenic gene expression. On the other hand, these changes were partially mitigated in the DOX + free FA group. Notably, FA-NIO treatment reduced histopathological damage, normalised reproductive hormone levels, markedly increased steroidogenesis-related gene expression, suppressed apoptotic and inflammasome-related gene expression, and greatly restored antioxidant capacity. Compared with DOX + free FA, FA-NIO consistently demonstrated greater protective efficacy. In summary, FA-NIO targets oxidative stress-mediated apoptosis, attenuates NLRP3 inflammasome-related signaling, and restores steroidogenic function, thereby successfully attenuating DOX-induced testicular toxicity. These results suggest that FA-NIO may represent a promising nanotherapeutic strategy for attenuating chemotherapy-induced reproductive toxicity in a rat model.

## Introduction

Infertility is a major global health issue that affects roughly 8–12% of partners worldwide [70, 71]. The World Health Organisation (WHO) defines infertility as the inability to conceive following a minimum of 12 months of consistent, unprotected sexual activity [74]. Male-related factors contribute solely to nearly 20–30% of infertility cases and are involved in up to 50% of affected couples [21]. Male infertility results from various idiopathic, acquired, and congenital causes. Regarding acquired factors, they are affected by chronic illnesses and treatments such as radiation, chemotherapy, and cancer [7].

Chemotherapeutic agents are widely recognized for their deleterious effects on male reproductive function and spermatogenesis [26]. Among these agents, doxorubicin (DOX), an anthracycline antibiotic composed of an aglycone tetracyclic ring structure linked to the amino sugar daunosamine, remains one of the most commonly used anticancer drugs for the treatment of solid tumors and hematological malignancies, including breast, prostate, ovarian, gastric, hepatic, and childhood cancers, as well as leukemias and sarcomas [54]. Despite its efficacy, DOX administration is associated with marked reproductive toxicity manifested by oxidative stress, inflammatory activation, and apoptosis of testicular cells.

Growing evidence suggests that reactive oxygen species (ROS) overproduction brought on by DOX is a major factor in testicular inflammation by activating nuclear factor kappa B (NF-κB) signaling pathways [69]. ROS also serve as critical triggers for activation of the NLR family pyrin domain-containing 3 (NLRP3) inflammasome, a cytosolic protein complex responsible for initiating inflammatory cascades [75]. NLRP3 activation leads to caspase-1 cleavage and the subsequent maturation and secretion of pro-inflammatory cytokines interleukin-1β (IL-1β) and interleukin-18 (IL-18) [39]. This process promotes NLRP3 inflammasome-related signaling, a highly inflammatory form of programmed cell death contributing to tissue injury and impaired spermatogenesis [41].

Natural products and herbal medicines derived from plant components have gained increasing attention for infertility management due to their safety profiles and biological efficacy [34]. Ferulic acid (FA), chemically known as *(E)-3-(4-hydroxy-3-methoxyphenyl)prop-2-enoic acid*, is one of the most abundant plant-derived phenolic acids and is commonly found in foods such as wheat bran, corn bran, artichokes, beans, and eggplant [64]. FA exhibits potent antioxidant, anti-inflammatory, anticancer, antidiabetic, and antimicrobial activities [73]. Notably, FA has demonstrated protective actions on the male reproductive system, improving sperm motility, viability, and counts in diabetic experimental models [57]. Its strong antioxidant properties stem from its ability to scavenge free radicals, inhibit lipid peroxidation, suppress ROS and reactive nitrogen species generation, and stabilize phenoxy radical intermediates through hydrogen donation from its hydroxyl functional groups [19].

Despite its promising therapeutic potential, the clinical utility of FA is markedly constrained by poor aqueous solubility and chemical instability, which compromise its bioavailability and pharmacological efficacy [62]. Although FA has demonstrated protective effects against oxidative stress and reproductive dysfunction, its effectiveness in mitigating DOX-induced testicular injury remains inadequately explored, particularly in relation to NLRP3 inflammasome-related signaling. Moreover, conventional FA administration does not ensure efficient delivery to target tissues, thereby reducing its clinical applicability.

Encapsulation strategies using nanocarrier systems have been widely employed to overcome these limitations and improve drug stability and delivery efficiency [11]. Among these systems, niosomes, a vesicular bilayer system composed of cholesterol and non-ionic surfactants, enhance drug solubility, dissolution, stability, half-life, and controlled release of medications to target organs[42]. However, the potential of FA-loaded niosomes (FA-NIO) in attenuating DOX-induced testicular toxicity has not been previously elucidated. Therefore, the present study was designed to investigate, for the first time, the protective effects of FA-NIO against DOX-induced testicular damage in rats, with a particular focus on oxidative stress, apoptosis, NLRP3 inflammasome activation, and steroidogenesis disruption.

## Materials and methods

### Chemicals

We obtained ferulic acid from Sigma-Aldrich, located in St. Louis, MO, USA. Doxorubicin hydrochloride was supplied by Alpha Chimica (MH, India). Sorbitan monostearate (Span 60) and cholesterol were purchased from Sigma-Aldrich (St. Louis, MO, USA). Methanol and chloroform were purchased from El-Gomhorea Chemical Company, Egypt. The purity of all other chemicals, solvents, and reagents was the highest.

### Experimental animals and ethical considerations

We obtained male albino Wistar rats aged 10 weeks old, weighing 250–260 g, from the laboratory animal farm at the Faculty of Veterinary Medicine, Zagazig University. Each rat was housed in a stainless-steel cage maintained at a temperature between 21 and 24 °C, providing a clean, pathogen-free environment. To achieve ideal living circumstances, the rats were exposed to a 12-h light–dark cycle and a 60% relative humidity. All animal experiments were performed in compliance with the ARRIVE guidelines and in accordance with the U.K. Animals (Scientific Procedures) Act, 1986, EU Directive 2010/63/EU, and the National Institutes of Health Guide for the Care and Use of Laboratory Animals (NIH Publications No. 8023, revised 1978). The ethics committee of the Faculty of Veterinary Medicine at Zagazig University, Egypt, examined and approved the research protocol (approval number: ZUIACUC/2/F/191/2025). All procedures were carried out in compliance with the relevant laws and regulations. The authors declare, under their legal and civil personal responsibility, that these regulations and guidelines were strictly followed.

### Preparation of FA-NIO by thin film hydration technique

The thin film hydration technique was used for preparing FA-NIO as previously described [30, 59]. The total lipid content 200 μmoles (span 60 and cholesterol) were added in an equimolar ratio (1:1). Briefly, in a round-bottom flask, span 60, cholesterol, and FA were dissolved in 10 ml of a chloroform: methanol solvent mixture (2:1). Then a reduced pressure was applied to evaporate the organic solvent and form a thin layer on the flask’s wall using a rotary evaporator (Heidolph-2 rotary evaporator, Germany) rotating at 150 rpm for 30 min at 60 °C. The flask was placed in a desiccator under vacuum for a night to eliminate any remaining solvent. After that, the hydration of the dried film was done using 10 ml of phosphate buffer solution (PBS, pH 6.8) at 60°C for one hour with a gentle agitation using the rotary evaporator to produce a milky dispersion of the FA-NIO.

### In vitro evaluation of FA-NIO

For characterizing the FA-NIO, several in vitro evaluation tests, including particle size, polydispersity index (PDI), zetapotential, transmission electron microscope (TEM), entrapment efficiency (EE%), and in vitro release study, were conducted.

#### Particle size (PS), polydispersity index (PDI), and zeta potential (ZP)

Dynamic light scattering (DLS) (Zetasizer Nano ZN, Malvern Panalytical Ltd, Malvern, UK) was used for measuring particle size, PDI, and zetapotential of FA-NIO formulation at a constant angle of 173° and a temperature of 25 °C. A predetermined volume of FA-NIO formulation was diluted with PBS (pH 6.8) (100X) and thereafter introduced into a transparent disposable zeta cell. The results were reported as mean values ± standard deviation (SD).

#### Transmission electron microscope (TEM)

Transmission electron microscope (Model JEM-1230, JOEL, Tokyo, Japan) was used for investigating the interior structure of the FA-NIO formulation, as mentioned previously [14]. In brief, a few drops of the formulation were placed on a carbon-coated grid and allowed to sit for 2 min to enhance their adherence to the carbon film. Any surplus liquid was blotted away with filter paper, and a drop of 1% phospho-tungstic acid was applied.

#### Entrapment efficiency (EE%)

For determining the EE%, FA-NIO was centrifuged for 1 h at 4 °C at 15,000 rpm, and then the supernatant was analyzed for the free ferulic acid using UV spectroscopy (Thermo Scientific Spectronic GENESYS 10 UV, Waltham, MA, USA) at λmax 310 nm [52]. The EE (%) was calculated by using the following equation:

EE (%) = (Total amount of ferulic acid-Free amount of ferulic acid)/(Total amount of ferulic acid) × 100.

#### In vitro cumulative release study

In vitro release study of ferulic acid from the niosomal system was conducted over 24 h using the dialysis bag technique [10]. In brief, niosomal pellets equivalent to 2 mg of ferulic acid were placed in a previously soaked dialysis bag. After that, the bags were immersed in a preheated receptor medium (37 °C) (pH 1.2) for 2 h, and then the bags were transferred to another preheated receptor medium (pH 6.8) for 22 h while being stirred at 100 rpm using a thermostatic shaking water bath. Samples of the receptor medium were collected at different time intervals and analyzed using UV spectroscopy at λmax 310 nm to measure ferulic acid absorbance. The volume of the receptor medium was restored using an equivalent volume of PBS maintained at 37 °C. To maintain the sink condition, an alcoholic receptor medium was used with a volume ratio of 80:20 PBS and methanol [20].

### Animals and experimental design

A total of forty male rats were randomly divided into four groups (n = 10 per group): control, DOX, DOX + FA, and DOX + FA-NIO. The control group (group I) received 0.5 mL of normal saline orally once daily for 21 days. The remaining 30 rats were administered doxorubicin (DOX) intraperitoneally at a dose of 3 mg/kg body weight on days 7, 14, and 21 [36], and were equally divided into three groups. The first group was the DOX group (group II), which received only DOX. The second group was the DOX + FA group (group III), which received DOX along with ferulic acid (FA) administered orally at 20 mg/kg body weight daily for 21 days, as reported by Sudheer et al. [66]. The third group was the DOX + FA-NIO group (group IV), which received DOX along with ferulic acid-loaded niosomes (FA-NIO) administered orally at 20 mg/kg body weight daily for 21 days [32]. The selected doses were based on prior literature reporting effective induction of toxicity (for DOX) and therapeutic efficacy (for FA and FA-NIO) in comparable animal models [32, 36, 66]. Following acclimatization, animals were assigned to the experimental groups according to the generated random sequence to ensure unbiased allocation. Blinding was applied during all outcome assessments that were amenable to masking, including histopathological examination, immunohistochemical scoring, and morphometric analyses. For these assessments, tissue samples and microscope slides were coded by an independent investigator who was not involved in data evaluation, and the investigators performing the analyses were unaware of group identities until all measurements were completed. Biochemical assays and molecular analyses (e.g., qRT-PCR) were conducted using coded samples whenever feasible; however, complete blinding was not always possible during sample preparation and data processing because of practical laboratory constraints.

### Sampling

Rats were anaesthetised after the study period. Anesthesia was induced via intraperitoneal injection of xylazine (5 mg/kg b.wt.) and ketamine hydrochloride (50 mg/kg b.wt.). After drawing blood from the medial canthus and centrifuging it in test tubes free of anticoagulants, a serum sample was utilised for biochemical examination. One part of the testis was dissected for gene expression (RT-qPCR). The second part was homogenized for oxidative stress assessment, and the other testis was used for histopathological and immunohistochemical analysis.

### Measurement of serum reproductive hormones

Serum samples were analyzed using rat-specific enzyme-linked immunosorbent assay (ELISA) kits to quantify hormone levels, including follicle-stimulating hormone (FSH) (Catalog No. E-EL-R0391, Texas, USA), luteinizing hormone (LH) (Catalog No. E-EL-R0026, Texas, USA), and testosterone (Catalog No. ab285350, Cambridge, United Kingdom).

### Sperm analysis

The cauda epididymis of the right testis was used to investigate sperm parameters. The epididymis was removed and put into a sterilized Petri dish with two milliliters of preheated normal saline and cut with microscissors to release the sperm. The Petri dishes were incubated at 37 °C for 5 min to allow the sperm to swim out of the epididymis. Sperm analysis (sperm motility, count, and morphology) was carried out according to the guidelines of the World Health Organization (WHO) [17]. All solutions and utensils were preheated at 37 °C before use.

#### Sperm motility

To evaluate sperm motility, a drop of semen sample was placed on a clean, dry glass slide, covered with a cover slip, and examined under both the low-power and high-power objectives of a light microscope (low power for mass motility of sperms and high power for individual motility of sperms). The average motility percentage was recorded in different microscopical fields.

#### Sperm morphology

Eosin-nigrosine-stained smears were prepared to assess the percentage of sperm abnormalities by using an oil immersion lens of a light microscope.100 spermatozoa were examined for each animal.

#### Sperm cell concentration

The epididymal suspension was added to Physiological saline solution at a ratio of 1:4, with the addition of five drops of formalin 40% to immobilize the spermatozoa. The number of spermatozoa /ml of semen was determined by using an improved Neubauer hemocytometer. The sperm cell concentration per ml of semen = sperm number in five secondary squares * dilution factor * 0.05*10^6^.

### Testicular oxidants/antioxidants status

Malondialdehyde (MDA) levels were determined using the thiobarbituric acid reactive substances (TBARS) assay according to[49], based on the formation of a pink chromogen measured spectrophotometrically at 532 nm. Results were expressed as nmol MDA/g tissue (Biodiagnostic, Giza, Egypt, CAT. No. 2529). Reduced glutathione (GSH) levels in testicular tissue were determined using the method of Abd El-Emam et al. [1], which relies on the reaction of GSH with 5,5′-dithiobis-(2-nitrobenzoic acid) (DTNB) to produce a yellow-colored product measured at 412 nm. GSH levels were expressed as nmol/mg protein (Biodiagnostic, Giza, Egypt, CAT. No. 2511). The technique introduced by Huber et al. [29] was used to measure the levels of SOD activity, based on the enzyme’s ability to inhibit the autoxidation of pyrogallol. The absorbance was recorded at 420 nm, and activity was expressed as U/mg protein (Biodiagnostic, Giza, Egypt, CAT. No. 2521). Catalase activity in testicular tissue was estimated using the method of Aebi [4], which measures the decomposition rate of hydrogen peroxide (H₂O₂) at 240 nm. Catalase activity was expressed as U/mg protein (Biodiagnostic, Giza, Egypt, CAT. No. 2517).

#### Real-time quantitative PCR (RT-qPCR) analysis

Previous documentation of the real-time analysis was done by Ibrahim et al. [31] and Mansour et al. [44]. Total RNA was extracted from testicular tissue using the ABT Total RNA Extraction Kit (Applied Biotechnology, Cat. No. ABT002, Cairo, Egypt) following the manufacturer’s instructions. RNA purity and concentration were evaluated using a NanoDrop spectrophotometer (Thermo Scientific, USA). Complementary DNA (cDNA) was synthesized from 1 µg of total RNA using the ABT 2 × RT Mix Oligo Kit (Applied Biotechnology, Cat. No. AMP11, Cairo, Egypt). Quantitative real-time PCR (qRT-PCR) was performed using the Applied Biosystems StepOne™ Real-Time PCR System. Amplification reactions (20 µL total volume) contained 10 µL of ABT 2 × qPCR Mix (SYBR) High ROX (Cat. No. AMP04, Applied Biotechnology, Ismailia, Egypt), 1 µL of forward primer (10 µM), 1 µL of reverse primer (10 µM), 2 µL of cDNA template, and 6 µL nuclease-free water. The sequences of all primers used are listed in Table 1. Thermal cycling conditions were as follows: initial denaturation at 95 °C for 10 min, followed by 45 cycles of 95 °C for 15 s, 59 °C for 40 s, and 72 °C for 40 s. Melting curve analysis was performed at the end of amplification to verify the specificity of the PCR products. The housekeeping gene GAPDH was used to normalize gene expression. Relative quantification of target genes was calculated using the 2^ − ΔΔCt method. [40]. All reactions were run in triplicate to ensure reproducibility.

**Table 1:** The sequence of primers used for RT-PCR

Gene	forward primer	reverse primer	Size (Pb)	accession no
NLRP3	TCTGTTCATTGGCTGCGGAT	TAGCCGCAAAGAACTCCTGG	189	NM_001191642.1
IL-1β	AGCTTCAGGAAGGCAGTGTC	ATCCCACGAGTCACAGAGGA	150	NM_031512.2
Bax	AGACGGCAACTTCAACTGGG	TGTTAAGCTAGGCCTGAGAGGT	199	XM_032893644.1
Bcl-2	TGTGGAGAGCGTCAACAGG	CAGAGACAGCCAGGAGAAATCA	178	NM_016993.2
StAR	ACACACTTTGGGGAGATGCC	AGCCACGTGAGTTTGGTCTT	116	XM_032918521.1
CYP11A1	CAGCGATGACCTATTCCGCT	AGGCATGTTGAGCATGGGAA	160	NM_017286.3
3β-HSD	TCAGCCGGACACTGGAAAAG	ATGACTTGGGAGCAGGTTGG	194	NM_054007.1
GAPDH	GGCACAGTCAAGGCTGAGAATG	ATGGTGGTGAAGACGCCAGTA	143	NM_017008.4

#### Immunohistochemical staining

Consecutive 4–5 µm-thick testicular tissue sections were deparaffinized in xylene and rehydrated through a graded series of ethanol. Antigen retrieval was performed by heating sections in citrate buffer (10 mM, pH 6.0) at 95 °C for 20 min. Endogenous peroxidase activity was quenched using 3% hydrogen peroxide for 10 min. Non-specific binding was blocked with 5% normal goat serum for 30 min at room temperature. For detection of protein expression, sections were incubated overnight at 4 °C with the following primary antibodies: recombinant anti-NLRP3 antibody (Abcam, Cambridge, UK, Cat. No. ab283819; 1:200), recombinant monoclonal caspase-1 antibody (Abcam, Cat. No. ab238972; 1:150), and anti-BAX antibody (Abcam, Cat. No. ab182734; 1:250). After washing, sections were incubated with biotinylated secondary antibody for 30 min at room temperature, followed by the avidin–biotin-peroxidase complex (ABC) solution according to [27]. Immunoreactivity was visualized using 3,3′-diaminobenzidine (DAB) as the chromogen and counterstained with hematoxylin. For quantification, ten 40 × photomicrographs per rat (50 photomicrographs per marker per group) were randomly selected, and staining intensity and area were analyzed using the color deconvolution plugin in ImageJ software [60].

#### Histopathological examination

The testicles were excised and handled with caution to minimize any damage to the delicate seminiferous tubules, and five shallow piercings to the tunica albuginea were performed using a 21-gauge needle before immersion in modified Davidson’s solution for 24 h [38]. Next, the testicles were processed for paraffin infiltration and embedding, and 4–5 µm thick transverse tissue sections from the middle portion of the testicles were prepared, stained with hematoxylin and eosin [15], and examined microscopically. Histopathological alterations were assessed semi-quantitatively using a scoring system defined as: 0 (none), 1 (mild), 2 (moderate), and 3 (severe), as outlined by [25]. All sections were evaluated independently by two blinded examiners throughout the histological analysis.

#### Statistical analysis

Statistical analyses were performed using GraphPad Prism software (version 10.0.1). Data were analyzed by one-way analysis of variance (ANOVA), followed by Tukey’s post hoc test. Results are expressed as the mean ± standard error of the mean (SEM), and statistical significance was set at *p* < 0.05. Histopathological scores were analyzed using non-parametric tests using Kruskal–Wallis, followed by Dunn’s post hoc test. Outcome assessments and data analysis were conducted in a blinded manner wherever feasible, while treatment administration was not blinded due to experimental constraints. Data distribution was assessed using the Shapiro–Wilk test to verify normality before statistical analysis.

## Results

### Physicochemical characterization of FA-NIO

**Fig. 1:**
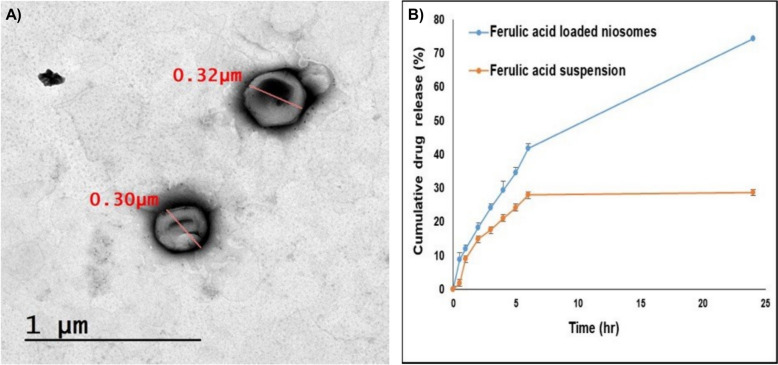
In vitro characterization of FA-NIO, **A** TEM of ferulic acid-loaded niosomes, and **B** in vitro drug release of ferulic acid-loaded niosomes compared with ferulic acid suspension

FA-NIO was successfully prepared using the thin-film hydration technique. Various in vitro nanocharacterizations, including particle size, PDI, zeta potential, EE%, and in vitro drug release, have been conducted. Our results showed an acceptable nano size for the ferulic acid-loaded nosome of 342 ± 35.17 nm with a homogenous distribution for the particles as indicated by the measured PDI of 0.25 ± 0.03. Moreover, these nanoparticles exhibited a negatively charged surface as indicated by their measured zeta potential of -44.47 ± 2.04. Transmission image Fig. 1A showed the spherical appearance of the niosomes with a size near that measured by Malvern Zetasizer. Additionally, the prepared niosomes showed a high entrapment for ferulic acid with an EE% of 75.77 ± 3.4%. Figure 1B showed the cumulative drug release percentage of ferulic acid-loaded niosomes compared with the FA suspension. After 6 h, 41.87% of ferulic acid was released from the prepared niosomes compared with the suspension, which showed a release of 27.96%; then after 24 h, the release from the niosomes reached 74.41% compared with 28.73% for the suspension, indicating the capability of the niosome nanoparticles to enhance the solubility of FA and, at the same time, achieve a sustained drug release.

### Sperm analysis results

**Fig. 2:**
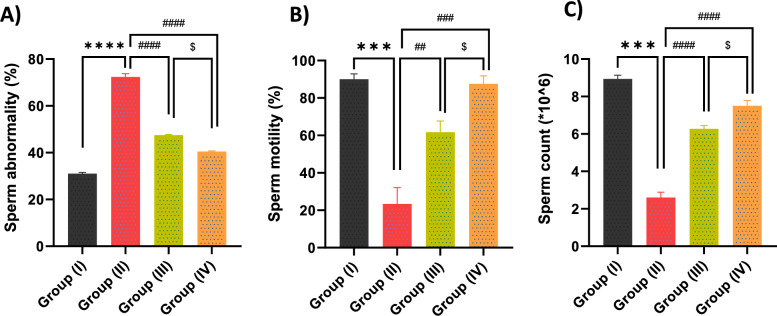
Protective effect of FA-NIO on semen parameters in DOX-induced reprotoxicity. **A** Sperm abnormalities percent, **B** Sperm motility percent, **C** Sperm cell concentration. Data are presented as mean ± SEM (n = 10). Statistical significance: ****P < 0.0001 vs Control, ***P < 0.001 vs control; ^####^P < 0.0001 vs DOX group, ^###^P < 0.001 vs DOX group, ^##^P < 0.01 vs DOX group; ^$^P < 0.05 vs DOX + FA group. Group (I), Control; Group (II), DOX-treated rats; Group (III), DOX + FA-treated rats; and Group (IV), DOX + FA-NIO-treated rats

**Fig. 3:**
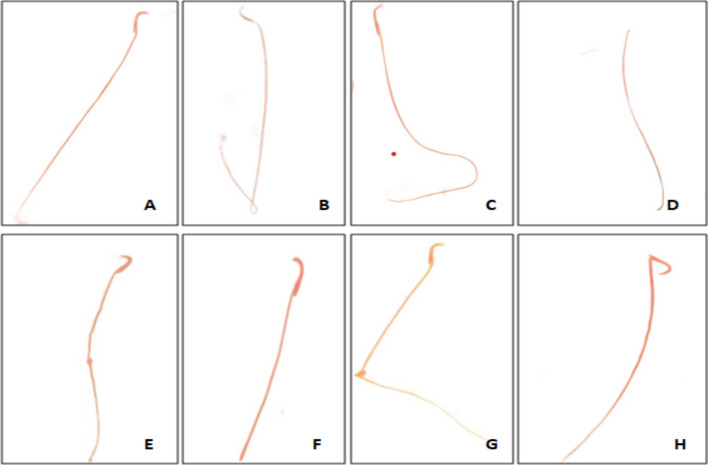
Morphological abnormalities of sperms in rats exposed to Doxorubicin treatment. **A** Normal sperm, **B** Looped tail, **C** Curved tail, **D** Detached head, **E** Distal protoplasmic droplet, **F** short tail, **G** Bent tail with distal protoplasmic droplet, and **H** Broken head. Photographs of various types of sperm abnormalities were evaluated in eosin–nigrosin–stained slides and viewed under the oil-immersion lens of a light microscope

Spermatological parameters (sperm abnormalities, motility, and semen count [106/mL]) are presented in Fig. 2A-C. The results showed notable variations in sperm parameters within the various experimental groups. While the percentage of abnormal sperm was substantially (P < 0.0001) higher in the DOX-treated group by 133.87% than in the control group, the percentage of abnormal sperm in the free FA and FA-NIO-treated groups appeared to be significantly(P < 0.0001) reduced by 34.48% and 44.13%, respectively, compared with the DOX-treated group. Furthermore, the FA-NIO-treated group had a substantially(P < 0.05) lower percentage of sperm abnormalities than the free FA group (Fig. 2A). As shown in Fig. 3 (A-H), many morphological abnormalities of sperm were identified in rats treated with DOX, including looped, curved, short, and bent tails, distal protoplasmic droplets, detached, and broken heads.

The sperm motility percentage was lower in the DOX-treated group by 77.77% than in the control group (P < 0.001). Rats treated with free or nano FA had substantial improvements in motility (%) by 200% and 337.5%, respectively, compared with those in the DOX-treated group (p < 0.01; p < 0.001, respectively). Moreover, the FA-NIO-treated group had a significantly(P < 0.05) higher motility percentage than the free FA-treated group (Fig. 2B).

A significant decline in sperm count was observed in the DOX-treated group by 70.94% compared with the control group (P < 0.001). An increase in sperm count was observed in both the free and nano FA groups by 142.30% and 188.46%, respectively, compared with the DOX group (p < 0.001). Furthermore, the FA-NIO-treated group had a significantly(P < 0.05) higher sperm count than the free FA-treated group (Fig. 2C).

The highest content of sperm motility, count, and normal morphology was recorded in the FA-NIO-treated group. These results further support the efficiency of using FA-NIO rather than free FA to improve the semen quality affected during DOX treatment.

### FA-NIO restores serum reproductive hormone homeostasis in DOX-induced testicular dysfunction

**Fig. 4:**
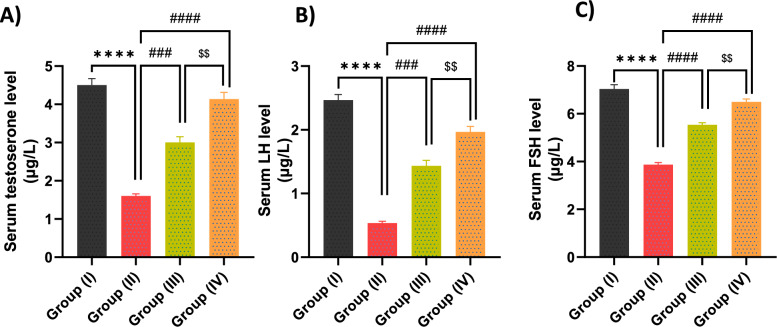
Effect of FA-NIO oral dosing on serum reproductive hormone levels. **A** testosterone level, **B** LH level, **C** FSH level. Data are presented as mean ± SEM (n = 10). Statistical significance: ****P < 0.0001 vs Control; ^####^P < 0.0001 001 vs DOX group, ^###^P < 0.001vs DOX group; ^$$^P < 0.01 vs DOX + FA group. Group (I), Control; Group (II), DOX-treated rats; Group (III), DOX + FA-treated rats; and Group (IV), DOX + FA-NIO-treated rats

Serum reproductive hormone levels were evaluated to investigate the protective potential of FA-NIO against DOX-induced reproductive dysfunction. DOX treatment markedly reduced serum testosterone levels by 0.6-fold and considerably lowered LH and FSH levels by 0.8- and 0.5-fold, respectively. In contrast, FA administration to DOX-treated rats significantly increased testosterone levels by 0.9-fold and increased LH and FSH levels by 1.7- and 0.4-fold, respectively Fig. 4A-C. Notably, FA-NIO treatment further improved reproductive hormone balance in DOX-exposed rats, resulting in a 1.5-fold increase in serum testosterone and a pronounced increase in LH and FSH levels by 2.7- and 0.7-fold, respectively Fig. 4A-C. Among all treatment groups, FA-NIO exerted the most substantial protective effect against DOX-induced reproductive dysfunction. Compared with FA treatment, FA-NIO further elevated testosterone levels by 0.3-fold and raised LH and FSH levels by 0.4- and 0.2-fold, respectively Fig. 4A-C.

### FA-NIO attenuates oxidative stress and enhances antioxidant defense in the testes of DOX-treated rats

**Fig. 5:**
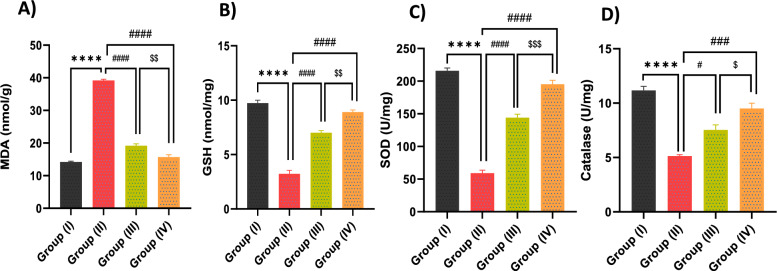
Effect of FA-NIO administration on oxidative stress markers in testicular tissues in DOX-rats. The levels of **a** MDA, **b** GSH, **c** SOD, and **d** catalase were estimated for the different groups. Data are presented as mean ± SEM (n = 10). Statistical significance: ****P < 0.0001 vs. control; ^####^P < 0.0001 vs DOX group, ^###^P < 0.001 vs DOX group, ^#^P < 0.05 vs DOX group;, ^$$$^P < 0.001 vs DOX + FA group, ^$$^P < 0.01 vs DOX + FA group, ^$^P < 0.05 vs DOX + FA group. Group (I), control; Group (II), DOX-treated rats; Group (III), DOX + FA-treated rats; and Group (IV), DOX + FA-NIO-treated rats

The antioxidant potential of FA-NIO was evaluated by measuring the levels of MDA, GSH, SOD, and catalase in rat testicular tissues. DOX treatment markedly increased MDA levels by 1.8-fold and significantly reduced GSH, SOD, and catalase levels by 0.6-, 0.7-, and 0.5-fold, respectively. In contrast, FA treatment in DOX-exposed rats significantly attenuated oxidative stress, as evidenced by a 0.5-fold reduction in MDA and increases in GSH, SOD, and Catalase levels by 1.2-, 1.4-, and 0.5-fold, respectively Fig. 4A-D. Notably, FA-NIO administration further enhanced the antioxidant status of testicular tissue in DOX-treated rats, resulting in a 0.6-fold decrease in MDA and marked elevations in GSH, SOD, and Catalase by 1.7-, 2.3-, and 0.8-fold, respectively Fig. 5A-D. Among all treatments, FA-NIO exhibited the most pronounced protective effect against oxidative stress. Compared with FA treatment alone, FA-NIO further reduced MDA levels by 0.2-fold and increased GSH, SOD, and catalase levels by 0.3-, 0.4-, and 0.3-fold, respectively, in DOX-treated rats Fig. 5A-D.

### FA-NIO suppresses NLRP3 inflammasome–associated gene expression in the testes of DOX-treated rats

**Fig. 6:**
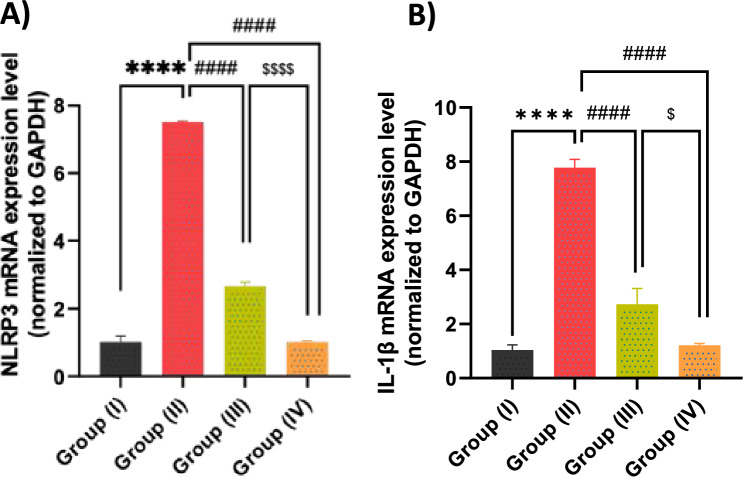
Effect of FA-NIO administration on mRNA expression of **A** NLRP3, **B** IL-1β in the testis of DOX-rats. Data are presented as mean ± SEM (n = 10). Statistical significance: ****P < 0.0001 vs control; ^####^P < 0.0001 vs DOX group;, P < 0.0001 vs DOX + FA group, ^$^P < 0.05 vs DOX + FA group. Group (I), control; Group (II), DOX-treated rats; Group (III), DOX + FA-treated rats; and Group (IV), DOX + FA-NIO-treated rats

To elucidate the molecular alterations underlying DOX-induced testicular injury and to evaluate the therapeutic efficacy of FA-NIO, the mRNA levels of NLRP3 and IL-1β were examined. DOX exposure resulted in a pronounced upregulation of NLRP3 expression, reaching approximately a six-fold increase, accompanied by a 6.5-fold elevation in IL-1β expression as opposed to the control group. Treatment of DOX-exposed rats with FA significantly downregulated NLRP3 and IL-1β expression to 0.6- and 0.65-fold, respectively, versus the DOX group. Similarly, FA-NIO administration markedly reduced NLRP3 and IL-1β expression to 0.9- and 0.8-fold, respectively, in DOX-treated rats. Notably, FA-NIO exerted a more potent inhibitory effect on NLRP3 and IL-1β expression, achieving reductions of 0.6- and 0.5-fold, respectively, compared with FA treatment alone (Fig. 6A, B).

### FA-NIO modulates apoptosis-related gene expression in the testes of DOX-treated rats

**Fig. 7:**
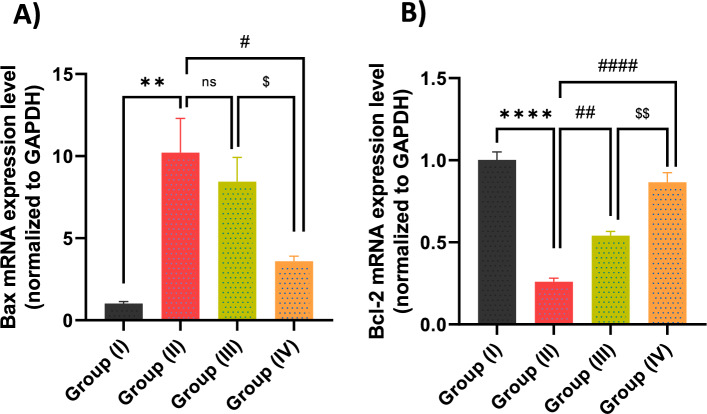
Effect of FA-NIO administration on the mRNA expression of apoptosis-related genes in testicular tissues in DOX-rats. Relative expression levels of **A** Bax, **B** Bcl-2 were quantified by qRT-PCR. Data are presented as mean ± SEM (n = 10). Statistical significance: ****P < 0.0001 vs Control, **P < 0.01 vs control; ^####^P < 0.0001 vs DOX group, ^##^P < 0.01 vs DOX group, ^#^P < 0.05 vs DOX group; ^$$^P < 0.01 vs DOX + FA group, ^$^P < 0.05 vs DOX + FA group; ns indicates non-significant. Group (I), control; Group (II), DOX-treated rats; Group (III), DOX + FA-treated rats; and Group (IV), DOX + FA-NIO-treated rats

DOX-treated rats demonstrated a marked upregulation of Bax mRNA expression by approximately nine-fold, while Bcl-2 expression was significantly downregulated to 0.7-fold compared with the untreated control group. In FA-treated rats, Bax mRNA levels were reduced to 0.2-fold, accompanied by a significant restoration of Bcl-2 expression to onefold relative to the DOX group. In contrast, FA-NIO treatment resulted in a more pronounced modulation of apoptotic markers, with Bax expression reduced to 0.6-fold and Bcl-2 significantly upregulated by 2.3-fold compared with DOX-treated rats. Overall, FA-NIO treatment yielded superior anti-apoptotic effects compared with FA alone. Relative to FA treatment, FA-NIO further decreased Bax mRNA levels by 0.5-fold and enhanced Bcl-2 expression by 0.6-fold (Fig. 7A, B).

### FA-NIO regulates the mRNA expression of steroidogenesis pathway–related genes in the testes of DOX-treated rats

**Fig. 8:**
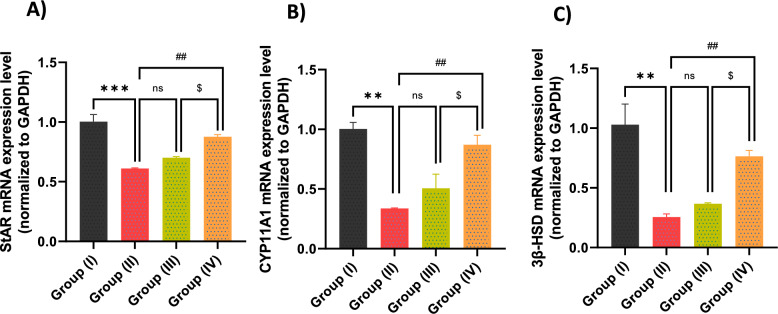
Effect of FA-NIO administration on the mRNA expression of steroidogenesis-related genes in testicular tissues in DOX-rats. Relative expression levels of **A** StAR, **B** CYP11A1, and **C** 3β-HSD were quantified by qRT-PCR. Data are presented as mean ± SEM (n = 10). Statistical significance: ***P < 0.001 vs Control, **P < 0.01 vs control, ^##^P < 0.01 vs DOX group; ^$^P < 0.05 vs DOX + FA group; ns indicates non-significant. Group (I), control; Group (II), DOX-treated rats; Group (III), DOX + FA-treated rats; and Group (IV), DOX + FA-NIO-treated rats

To clarify the molecular basis of DOX-induced testicular impairment and the protective efficacy of FA-NIO, the transcriptional expression of key steroidogenesis-related genes was analyzed in the testes of DOX-treated rats. DOX administration resulted in a marked downregulation of StAR, CYP11A1, and 3β-HSD, with expression levels reduced by 0.4, 0.6, and 0.7-fold, respectively, versus the control group. In contrast, treatment with FA non-significantly raised the expression of StAR, CYP11A1, and 3β-HSD by 0.2-, 0.5-, and 0.4-fold, respectively, relative to the DOX group. Notably, FA-NIO treatment produced a more pronounced upregulation of these genes, increasing StAR, CYP11A1, and 3β-HSD expression by 0.4-, 1.5-, and twofold, respectively, in DOX-exposed rats. Moreover, FA-NIO demonstrated superior efficacy compared with the FA group, further enhancing the expression of StAR, CYP11A1, and 3β-HSD by 0.2-, 0.7-, and 1.1-fold, respectively (Fig. 8A-C).

### Immunohistochemical analysis

**Fig. 9:**
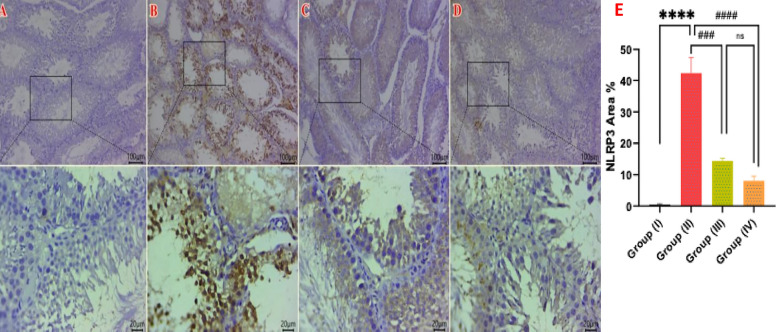
Photomicrographs of immunostained sections of testes for NLRP3 showing: no detectable immunopositive cells in group I (**A**). Strongly positive cytoplasmic-stained cells in abundant spermatogonia and spermatocytes at group II (**B**). Moderate numbers of weakly stained germinal cells at group III (**C**). Barely stained germinal epithelium within seminiferous tubules at group IV (**D**). Graphical demonstration of the area % of NLRP3 expression in the different groups (**E**). ****P < 0.0001 vs control, ^###^P < 0.001 vs DOX group, ^####^P < 0.0001 vs DOX group; ns indicates non-significant. IHC counterstaining with Mayer's haematoxylin. (The positive expressed cells revealed a golden brown color). Scale bars: 100 μm (upper panels, A–D) and 20 μm (corresponding enlarged lower panels). Group (I), control; Group (II), DOX-treated rats; Group (III), DOX + FA-treated rats; and Group (IV), DOX + FA-NIO-treated rats

Immunostained testicular sections for NLRP3 showed an absence of immunopositive cells in the control group (Fig. 9A). In contrast, the dox group exhibited strong cytoplasmic immunoreactivity in abundant spermatogonia and spermatocytes (Fig. 9B). Meanwhile, a marked reduction in immunostaining intensity was observed in both free ferulic acid and FA-NIO groups. Specifically, free ferulic acid (Fig. 9C) demonstrated a moderate number of weakly stained germinal cells, whereas the FA-NIO group displayed barely detectable immunostaining within the germinal epithelium of the seminiferous tubules (Fig. 9D).

**Fig. 10:**
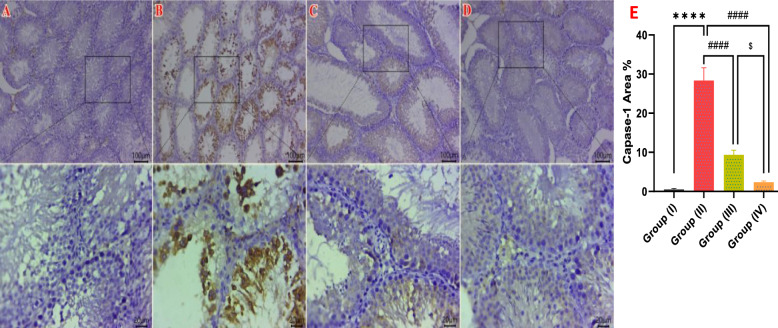
Photomicrographs of immunostained sections of testes for Caspase-1, showing negative immunopositive cells at group I (**A**). Numerous positive cytoplasmic-stained cells in the germinal epithelial lining of seminiferous tubules at group II (**B**). moderate numbers of weakly stained cells at group III (**C**). Faintly stained germinal epithelium at group IV (**D**). Graphical demonstration of the area % of Caspase-1 expression in the different groups (**E**). Statistical significance: ****P < 0.0001 vs control, ^####^P < 0.0001 vs DOX group; ^$^P < 0.05 vs DOX + FA group. IHC counterstaining with Mayer's haematoxylin. (The positive expressed cells revealed a golden brown color). Scale bars: 100 μm (upper panels, A–D) and 20 μm (corresponding enlarged lower panels). Group (I), control; Group (II), DOX-treated rats; Group (III), DOX + FA-treated rats; and Group (IV), DOX + FA-NIO-treated rats

Immunostained testicular sections for caspase-1 demonstrated negative immunoreactivity in the control group (Fig. 10A). However, the dox group (Fig. 10B) revealed numerous strongly positive cytoplasmic-stained cells within the germinal epithelium lining the seminiferous tubules. In contrast, free ferulic acid (Fig. 10C) showed a moderate number of weakly stained cells, while FA-NIO (Fig. 10D) exhibited faint immunostaining within the germinal epithelium.

**Fig. 11:**
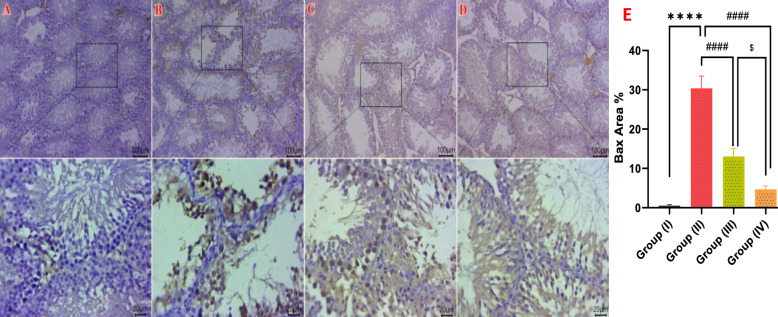
Photomicrographs of immunostained sections of testes for Bax showing: negative immunostained cells at group I (**A**), marked numbers of positive-stained germinal epithelium at group II (**B**). moderate numbers of positive-expressed germ cells at group III (**C**). Mild numbers of immuno-stained germinal cells at group IV (**D**). Graphical demonstration of the area % of Bax expression in the different groups (**E**). Statistical significance: ****P < 0.0001 vs control, ^####^P < 0.0001 vs DOX group; ^$^P < 0.05 vs DOX + FA group. IHC counterstaining with Mayer's haematoxylin. (The positive expressed cells revealed a golden brown color). Scale bars: 100 μm (upper panels, A–D) and 20 μm (corresponding enlarged lower panels). Group (I), control; Group (II), DOX-treated rats; Group (III), DOX + FA-treated rats; and Group (IV), DOX + FA-NIO-treated rats

Immunostained testicular sections for Bax revealed an absence of immunopositive cells in the control group (Fig. 11A). In contrast, the dox group (Fig. 11B) exhibited a marked increase in Bax immunoreactivity, with numerous positively stained germinal epithelial cells. Meanwhile, a gradual attenuation of Bax immunoexpression was observed in both free ferulic acid and FA-NIO groups. Specifically, free ferulic acid (Fig. 11C) showed a moderate number of Bax-positive germ cells, whereas the FA-NIO group (Fig. 11D) displayed only mild immunostaining in a limited number of germinal cells.

### FA-NIO ameliorates DOX-induced histopathological alterations in testicular tissue

**Fig. 12:**
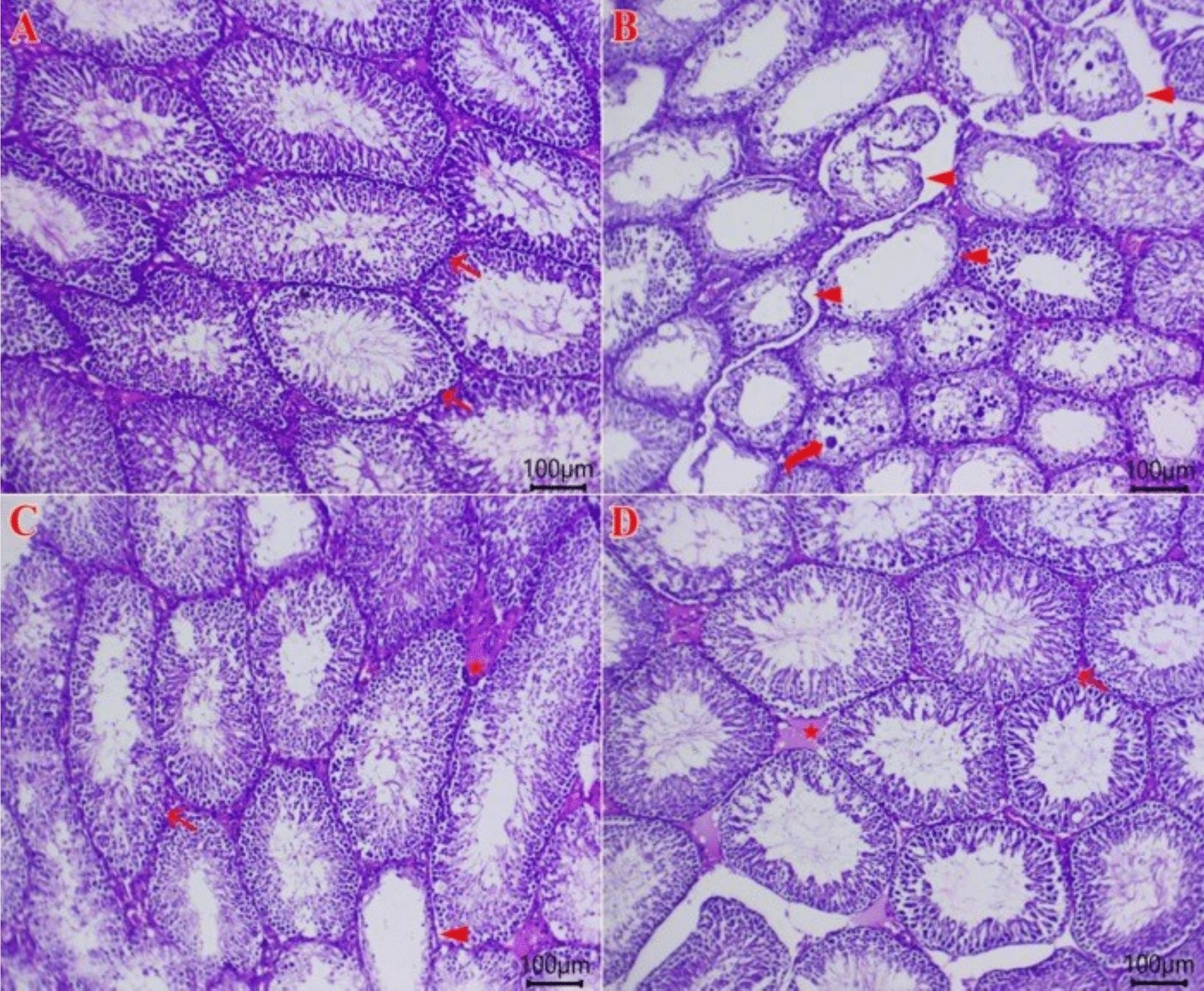
photomicrographs of H&E-stained sections of testes showing: normal histological structures of germinal epithelial lining seminiferous tubules (arrows) and Leydig cells in group I (**A**). Numerous atrophied seminiferous tubules (arrowheads) with necrotic germinal epithelium with pyknotic nuclei, empty most tubular lumina from sperms, and the presence of spermatocytes and giant cells (curved arrow) beside interstitial edema in group II (**B**). apparent normal most seminiferous tubules (arrow), few atrophied tubules with necrotic germ cells (arrowhead), and mild interstitial edema (star) at group III (**C**). Restoration of most histological structures of seminiferous tubules (arrow) with mild edema between tubules (star) in the group IV (**D**). (Scale bar 100μm). Group (I), control; Group (II), DOX-treated rats; Group (III), DOX + FA-treated rats; and Group (IV), DOX + FA-NIO-treated rats

Normal testicular histology was observed in the control group. Testes from control rats (Fig. 12A) exhibited intact seminiferous tubules with a normal germinal epithelial lining and well-defined Leydig cells. The seminiferous tubules contain orderly layers of spermatogonia, spermatocytes, spermatids, and centrally located mature spermatozoa. In contrast, testes from DOX-treated rats (Fig. 12B) showed marked histopathological alterations, including numerous atrophied seminiferous tubules characterized by necrotic germinal epithelium with pyknotic nuclei, largely empty tubular lumina devoid of sperm, the presence of giant spermatocyte cells, and interstitial edema between some tubules. Conversely, a gradual improvement in testicular histoarchitecture was observed in both the FA and FA-NIO-treated groups. The FA group (Fig. 12C) exhibited only a few atrophied seminiferous tubules with necrotic germ cells, accompanied by mild interstitial edema. Notably, the FA-NIO group (Fig. 12D) demonstrated substantial restoration of normal testicular histological features, with only mild edema observed between some seminiferous tubules. The scoring system for evaluating common observed lesions in testicular tissues among different groups is listed in Table 2.

**Table 2:** Lesion scores reflecting the severity and extent of testicular tissue damage were analyzed using the using the Kruskal–Wallis test followed by Dunn’s post hoc test

Lesion (Mean Rank Score)	Control	DOX	DOX + FA	DOX + FA NIO
Atrophied seminiferous tubules	0.0 (0.00)	2.6 (0.00)**	1.6 (1.00)#	0.2 (0.50)##†
Degenerated/necrotic germinal epithelium	0.0 (0.00)	2.8 (0.00)**	1.4 (1.00)#	0.4 (0.50)##†
Interstitial edema	0.0 (0.00)	2.0 (0.00)**	1.2 (1.00)#	0.6 (0.50)#††
Spermatocyte giant cells	0.0 (0.00)	2.2 (0.00)**	0.4 (0.50)##	0.0 (0.00)##††

Examined rats = 10 rats/group. Number of examined fields (10 random fields/group, 400X). Data are expressed as a mean score. **P < 0.01 vs control group. ^#^P < 0.05 vs DOX group. ^##^P < 0.01 vs DOX group. ^†^P < 0.05 vs DOX + FA group.^††^P < 0.01 vs DOX + FA group

## Discussion

The chemotherapeutic DOX, which is produced from anthracyclines, is frequently used to treat solid and hematological cancers [45]. However, clinical application is constrained by the harmful effects of DOX, especially on the heart and kidneys, and it also induces significant cytotoxicity in testicular tissue [53]. DOX induces testicular toxicity that impairs sperm quality, contributes to testicular atrophy, and ends up being infertility, ultimately reducing the quality of life of male cancer patients. These adverse effects are mainly driven by oxidative stress, inflammation, DNA damage, and apoptosis [6]. Doxorubicin-induced tissue toxicity is primarily mediated through excessive generation of reactive oxygen species (ROS), leading to oxidative stress, mitochondrial dysfunction, and subsequent cellular injury [35]. In the present study, the observed elevation in lipid peroxidation markers, along with concomitant depletion of key antioxidant defenses, strongly supports the establishment of a redox imbalance in affected tissues. This oxidative burden is known to disrupt membrane integrity, impair mitochondrial bioenergetics, and trigger apoptotic and necrotic pathways. Moreover, the histopathological alterations detected, including cellular degeneration, necrosis, and inflammatory cell infiltration, are consistent with the well-documented cytotoxic effects of doxorubicin, which are further amplified by inflammatory signaling cascades [24]. The interplay between oxidative stress and inflammation likely exacerbates tissue injury, creating a vicious cycle that promotes disease progression. Importantly, the amelioration of these biochemical and histological abnormalities following treatment suggests that the protective effect is mediated, at least in part, through attenuation of oxidative stress and suppression of inflammation, thereby preserving cellular architecture and function. The goal of the current investigation was to look into the potential underlying mechanisms and modulating effect of FA-NIO against DOX-induced testicular damage. According to the results of our investigation, FA-NIO, an enhanced formulation of FA, exhibits significant potential as a therapeutic strategy when compared to free FA due to its potent modulation of oxidative stress, apoptotic signaling, NLRP3 inflammasome-related signaling, and steroidogenic pathways. FA-NIO is more effective in preventing DOX-induced injury to testicular tissue compared to conventional FA.

Previous studies showed that free radicals have detrimental effects on the quality of spermatozoa, such as morphology, count, and motility [5]. Free radicals induce oxidative stress results in decreased sperm concentration, motility, and elevated sperm abnormalities [68]. Spermatozoa are more sensitive to oxidative stress as they have a small amount of cytoplasm, an inadequate level of antioxidants, and a high concentration of unsaturated fatty acids in their cytoplasm [16]. Furthermore, due to the unique structure of the sperm cell, internal antioxidant enzymes cannot adequately safeguard the plasma membrane across the acrosomal and tail regions [8]. Thus, sperm health and fertilizing capacity rely greatly on the antioxidant capacity of the seminal fluid. These antioxidants act as a primary defense mechanism, neutralizing ROS to prevent oxidative stress-induced injury to the spermatozoa [63]. In this study, a decrease in progressive motility, sperm count, and normal sperm morphology was observed in the group treated with DOX, which may be attributed to DOX-induced oxidative stress within the testes. Previous studies confirmed the toxic effect of DOX treatment on sperm parameters, as in the present study [9, 43]. Previous studies suggested that the decline in sperm count is due to the reduction of germ cells across different developmental stages, implying that oxidative stress induced by DOX metabolism is a major contributor to the observed oligospermia[33]. Also, the observed morphological defects in spermatozoa may be attributed to DOX-induced impairments in DNA integrity, highlighting the potent genotoxicity of DOX toward germ cells [67]. Seemingly, this greater rise in sperm cell concentration, motility, and normality in free FA-treated groups than in DOX-treated groups, as noticed in this study’s results, was produced by the antioxidant features of FA, specifically through the inhibition of lipid peroxidation. The findings from this study agree with the previous studies of Roy et al. [57], who reported that FA had an enhancing effect on sperm parameters in FA-treated diabetic rats, and Adey et al. [3], who reported the therapeutic effect of FA against 2-methoxyhydroxymandelic oxidative stress in the testes of rats. Low bioavailability, which is due to the poor permeability across biological barriers and low stability and solubility in water, is the major disadvantage of free FA. So, the use of nanoparticles enhances the efficacy of FA through improving bioavailability and water solubility [61]. Interestingly, according to the results, the highest sperm count, normal sperms, and motility were observed in the nano FA-treated group. This means that nano treatments improved the destructive effects of DOX on sperm morphology more than free FA treatment.

Our findings demonstrated that DOX markedly reduced FSH, LH, and testosterone levels, in agreement with previous studies reporting DOX-induced hormonal disruption and associated infertility [50, 55]. Appropriate Leydig cell physiology and Sertoli cell activity during spermatogenesis rely on the balance between FSH and LH, stimulated by GnRH. Previous findings indicate that a negative feedback loop affecting testosterone synthesis in Leydig cells may stem from a pituitary–testicular hormone imbalance [48]. The hormonal imbalance induced by DOX is attributed, in part, to alterations in the activity of steroidogenic enzymes. Our findings demonstrated that rats exposed to DOX had lower mRNA levels of StAR, CYP11A1, and 3β-HSD, which complies with earlier research [69]. The initial and limiting step in steroidogenesis is the transport of cholesterol from the outer to the inner mitochondrial membrane, permitted by Steroidogenic Acute Regulatory (StAR) protein. Following this, CYP11A1 oxidatively cleaves cholesterol to form pregnenolone. Pregnenolone is then further oxidized by CYP17 enzymes in the endoplasmic reticulum to produce various C-19 steroids. Additionally, the 3-hydroxyl group is oxidized by 3β-HSD to create androstenedione, which is subsequently reduced by 17βHAD to provide testosterone [46, 65]. Free FA administration partially restored reproductive hormone levels in DOX-treated rats [37] found that FA improved the declining levels of the reproductive hormones in cadmium-intoxicated rats. Park and Han [51] demonstrated that FA inhibited the testosterone-degrading enzymes in the rat liver homogenate. Notably, FA-NIO elicited a markedly greater upregulation of StAR, CYP11A1, and 3β-HSD expression than free FA, which was reflected in the pronounced restoration of circulating testosterone and gonadotropin levels. This enhanced efficacy may be attributed to the nano-vesicular delivery system, which improves FA bioavailability, thereby maximizing its antioxidant and endocrine-modulating actions. Niosomes have been shown to enhance drug stability, prolong systemic circulation, and facilitate targeted delivery to vulnerable tissues, including the testes [47].

DOX-induced oxidative stress is a key contributor to testicular injury, promoting lipid peroxidation, DNA fragmentation, and apoptotic cell death. DOX treatment markedly elevated testicular MDA levels while significantly reducing SOD, catalase, and GSH activities. These findings are in alignment with those reported by Asana et al. [13]. Nevertheless, co-treatment with FA-NIO boosted the levels of these antioxidants and lowered lipid peroxidation indicators, demonstrating its antioxidative efficacy. Numerous studies have shown strong evidence of FA nanoparticles' capacity to prevent oxidative damage. In a study by Essa et al. [23], FA nanoparticles showed stronger antioxidant effects than conventional FA, offering better defense against oxidative damage in hepatic tissues caused by acrylamide. Elevated levels of GSH, GPx, SOD, and CAT were observed, accompanied by lower concentrations of MDA [58]. Furthermore, another investigation revealed that the FA nano-formulation exhibits better performance than double the original form. In comparison to the free FA group, the FA nano-formulation group exhibited a notable increase in GSH and a decrease in MDA levels, as well as a decrease in oxidative status indicators in kidney tissue [75].

Emerging evidence directly implicates NLRP3 inflammasome-related signaling in testicular pathology associated with male infertility. For instance, experimental models of varicocele demonstrate activation of the NLRP3–caspase-1 axis in testicular and epididymal tissues, leading to increased IL-1β/IL-18 production, germ cell apoptosis, and impaired spermatogenesis [12]. Moreover, aluminum exposure induces significant testicular toxicity, primarily through oxidative stress and activation of proinflammatory pathways, including the NLRP3/caspase-1/IL-1β inflammasome. This inflammatory cascade promotes macrophage activation, germ cell damage, and disruption of testicular function, leading to impaired spermatogenesis and hormonal imbalance [22]. The present findings demonstrated that DOX exposure markedly upregulated the mRNA expression of NLRP3 and its downstream pro-inflammatory cytokine IL-1β, suggesting activation of inflammasome-related inflammatory signaling. This observation is consistent with accumulating evidence showing that reactive oxygen species overproduction from DOX treatment activates the NLRP3 inflammasome, leading to caspase-1 activation [56]. Previous research showed that the NLRP3 inflammasome-related signaling is marked by cytoplasmic swelling and plasma membrane rupture, primarily through mastermind D (GESTUM) or DME cleavage, resulting in the release of IL-1β and IL-18 [72]. IL-1β negatively affects Leydig cell steroidogenesis, resulting in decreased testosterone production and perpetuating chronic local inflammation that harms the spermatogenic niche [18]. To support the deleterious effect of DOX on seminiferous tubules, we found that DOX significantly increased the apoptotic marker (Bax) in the testicular tissue, whereas apoptotic marker (Bcl-2) expression decreased. Importantly, treatment with FA attenuated the DOX-induced overexpression of NLRP3 and IL-1β, reflecting its potent anti-inflammatory and antioxidant properties. [28] found elevated levels of NLRP3, caspase-1, IL-1β, and IL-18 in serum and skin tissues following 40 Gy radiation, indicating that FA gel might protect against radiation-induced skin damage by inactivating the NLRP3 inflammasome. FA-NIO administration had a more pronounced inhibitory effect on NLRP3 and IL-1β expression compared with free FA, highlighting the advantage of niosomal encapsulation. Moreover, niosomal delivery may facilitate intracellular FA accumulation, thereby more effectively attenuating the expression of inflammasome-associated and apoptotic markers.

Furthermore, histological examination of testicular tissue from DOX-treated rats revealed extensive degeneration of seminiferous tubules, necrosis of germinal epithelium, pyknotic nuclei, empty tubular lumina devoid of spermatozoa, and the presence of giant spermatocyte cells, together with interstitial edema. These structural abnormalities are characteristic hallmarks of DOX-induced testicular toxicity and reflect severe impairment of spermatogenesis and Leydig cell function [2]. Notably, FA-NIO demonstrated the most pronounced histological recovery, with near-normal seminiferous tubule architecture and only minimal interstitial edema. This substantial structural restoration closely paralleled the robust recovery of steroidogenic gene expression and the marked elevation of circulating testosterone and gonadotropins.

## Conclusion

The study demonstrates that doxorubicin causes significant testicular toxicity through mechanisms like oxidative stress, upregulation of NLRP3 and related inflammatory markers, disrupted apoptotic signaling, and affected steroidogenic pathways. Treatment with FA provided partial protection, while niosomal encapsulation of FA significantly boosted its efficacy. FA-NIO restored antioxidant defenses, suppressed expression of NLRP3 inflammasome-related inflammatory markers, balanced apoptotic markers, and reinstated steroidogenic regulators, leading to better histological integrity and normalized hormone levels. This underscores the potential of FA-NIO as a nanotherapeutic approach for mitigating chemotherapy-induced testicular dysfunction in a preclinical setting and calls for further studies to evaluate its safety, efficacy, and possible clinical relevance. A limitation of the present study is the absence of additional control groups receiving ferulic acid (FA) alone, FA-loaded niosomes (FA-NIO) alone, and empty niosomes without the active compound. Although no overt adverse effects were observed in the treated groups, inclusion of these controls would have provided a more comprehensive assessment of the intrinsic biological effects of FA, the potential contribution of the niosomal carrier itself, and any interactions between the formulation components and normal testicular physiology. Future studies incorporating these additional control groups are warranted to better define the independent and synergistic contributions of the active compound and its nanocarrier.

## Data Availability

The corresponding author can provide the datasets used and/or analysed during the current investigation upon reasonable request.
